# Retinoic Acid Therapy Resistance Progresses from Unilineage to Bilineage in HL-60 Leukemic Blasts

**DOI:** 10.1371/journal.pone.0098929

**Published:** 2014-06-12

**Authors:** Holly A. Jensen, Rodica P. Bunaciu, Christopher N. Ibabao, Rebecca Myers, Jeffrey D. Varner, Andrew Yen

**Affiliations:** 1 School of Chemical and Biomolecular Engineering, Cornell University, Ithaca, New York, United States of America; 2 Department of Biomedical Sciences, Cornell University, Ithaca, New York, United States of America; 3 Department of Biology, Cornell University, Ithaca, New York, United States of America; 4 Department of Biology, Rutgers University, New Brunswick, New Jersey, United States of America; Emory University, United States of America

## Abstract

Emergent resistance can be progressive and driven by global signaling aberrations. All-*trans* retinoic acid (RA) is the standard therapeutic agent for acute promyelocytic leukemia, but 10–20% of patients are not responsive, and initially responsive patients relapse and develop retinoic acid resistance. The patient-derived, lineage-bipotent acute myeloblastic leukemia (FAB M2) HL-60 cell line is a potent tool for characterizing differentiation-induction therapy responsiveness and resistance in t(15;17)-negative cells. Wild-type (WT) HL-60 cells undergo RA-induced granulocytic differentiation, or monocytic differentiation in response to 1,25-dihydroxyvitamin D_3_ (D_3_). Two sequentially emergent RA-resistant HL-60 cell lines, R38+ and R38-, distinguishable by RA-inducible CD38 expression, do not arrest in G1/G0 and fail to upregulate CD11b and the myeloid-associated signaling factors Vav1, c-Cbl, Lyn, Fgr, and c-Raf after RA treatment. Here, we show that the R38+ and R38- HL-60 cell lines display a progressive reduced response to D_3_-induced differentiation therapy. Exploiting the biphasic dynamic of induced HL-60 differentiation, we examined if resistance-related defects occurred during the first 24 h (the early or “precommitment” phase) or subsequently (the late or “lineage-commitment” phase). HL-60 were treated with RA or D_3_ for 24 h, washed and retreated with either the same, different, or no differentiation agent. Using flow cytometry, D_3_ was able to induce CD38, CD11b and CD14 expression, and G1/G0 arrest when present during the lineage-commitment stage in R38+ cells, and to a lesser degree in R38- cells. Clustering analysis of cytometry and quantified Western blot data indicated that WT, R38+ and R38- HL-60 cells exhibited decreasing correlation between phenotypic markers and signaling factor expression. Thus differentiation induction therapy resistance can develop in stages, with initial partial RA resistance and moderate vitamin D_3_ responsiveness (unilineage maturation block), followed by bilineage maturation block and progressive signaling defects, notably the reduced expression of Vav1, Fgr, and c-Raf.

## Introduction

For three decades, retinoic acid (RA) differentiation therapy has been tantamount to transforming acute promyelocytic leukemia (APL) from a fatal diagnosis into a manageable disease. RA induces remission in 80–90% of APL PML-RARα-positive patients [1]. However, remission is not durable and relapsed cases exhibit emergent RA resistance [2], [3]. Meanwhile similar success stories have yet to be achieved for other cancer cell types. Parallel to the clinical use of RA in APL treatment, intense research has focused on understanding the source of cancer treatment relapse, and exploring the effectiveness of RA in other cancers.

Historically RA resistance in APL has been associated with mutation(s) in the PML-RARα fusion protein, rendering it unresponsive to RA. However, in some APL patients, PML-RARα mutations emerge months after termination of RA therapy, suggesting the existence of other defects [4]. In the patient-derived APL cell line NB4, RA resistance may or may not be correlated with mutant PML-RARα [4]. RA-resistant NB4 cells often remain partially RA-responsive in that they can upregulate RA-inducible differentiation markers, such as CD38 or CD18 [5]. HL-60, another patient-derived leukemia cell line, does not harbor the t(15;17) translocation pathognomonic for APL and thus lacks PML-RARα, but is nevertheless RA-responsive. Like NB4 cells, *in vitro* maturation of HL-60 cells is consistent with that of primary APL cells in culture and with clinical RA differentiation therapy progression [4]. Ectopic expression of RARα in RA-resistant HL-60 cells in which mutant RARα was found also does not necessarily restore RA responsiveness, again suggesting the presence of other defects [6], [7].

There is great interest in employing differentiation-promoting agents in combination with RA treatment to overcome resistance, and improve therapy and prognosis in APL and other cancer types. The active form of vitamin D_3_, 1,25-dihydroxyvitamin D_3_ (D_3_), which acts through vitamin D receptor (VDR), is capable of inducing differentiation in myelo-monocytic precursor cells, but has been less widespread as a clinical treatment since D_3_ also induces hypercalcemia and hyperphosphatemia. However, co-administration of RA with D_3_ is a potential therapeutic strategy to mitigate the side effects and limitations of each individual inducer. Bipotent human acute myeloblastic leukemia (FAB M2) HL-60 cells can be induced to terminally differentiate *in vitro* along the granulocytic lineage toward neutrophil-like cells using RA, while differentiation along the monocytic lineage can be achieved with D_3_.

RA-treated HL-60 undergoing granulocytic differentiation display early increased surface expression of CD38, followed by CD11b expression. D_3_-treated HL-60 cells undergoing monocytic differentiation express CD38, higher levels of CD11b, and the monocytic surface marker CD14. Induced terminal differentiation is accompanied by G1/G0 cell cycle arrest, and the development of inducible oxidative metabolism (respiratory burst), a function of mature granulocytes and monocytes. For the RA-treated case, differentiation requires sustained activation of mitogen-activated protein kinase (MAPK) signaling along the Raf/MEK/ERK axis [8], and a cascade of signaling regulatory events involving a putative signalosome containing c-Cbl, Vav1, and the Src-family kinases Lyn and Fgr [9], [10]. This is due in part to retinoic acid response elements (RAREs) in the promoter regions of CD38 and BLR1 [11], [12]. Both of these proteins are rapidly upregulated by RA; CD38 is the nexus for the putative signalosome while BLR1 drives a prolonged MAPK signal though its relationship with c-Raf [12]. However, D_3_-induced differentiation also requires sustained MAPK signaling [13] and results in upregulation of CD38 and CD38-associated factors.

Onset of G1/G0 arrest and terminal differentiation is slow requiring approximately 48 h of treatment, during which HL-60 cells undergo two sequential, functionally discernible stages [14]–[17]. With a doubling time of approximately 20–24 h, induced HL-60 cells first become primed for differentiation (precommitment phase) and undergo early differentiation events. During the subsequent 24 h, HL-60 complete a second cell division that results in terminally differentiating cells which are committed to a specific lineage determined by the inducer present, e.g. RA or vitamin D_3_ [14], [16]. Although lineage-specific events, such as CD14 expression, can in fact occur during the first 24–48 h of D_3_ treatment in HL-60 [13], the final inducer present is nonetheless the determining factor for lineage selection and subsequent terminal differentiation into that lineage [18]. It has also been shown that HL-60 cells treated with RA for 24 h followed by washing and no retreatment results in a still-proliferating population that retains a “memory” for differentiation that lasts 4–6 cell divisions [15]. During this time, cells proliferate until retreatment in which short RA doses can induce complete granulocytic differentiation.

We previously isolated two emergent RA-resistant HL-60 cell lines [19] after chronic RA exposure. These RA-resistant lines do not express CD11b, exhibit G1/G0 arrest, nor develop oxidative metabolism after RA treatment. One resistant line (R38+) retains RA-inducible CD38 expression while the other (R38-) has lost this ability. The R38- line, which sequentially emerged from R38+, thus appears to have an earlier defect which blocks the RA-induced differentiation sequence before the expression of CD38. Signaling events that define the wild-type response are compromised in both R38+ and R38-, which include RA-induced c-Raf expression and phosphorylation, c-Cbl and Vav1 expression, expression of Src-family kinases (SFKs) Lyn and Fgr and Y416 SFK phosphorylation.

In this study we examined whether the RA resistance defect segregates with lineage specificity, or with early or late stages of induced differentiation. An early defect might compromise both lineages, whereas a late defect might only affect the granulocytic lineage. Here we report that an RA-resistant cell line that retains partial RA-responsiveness (R38+) is more amenable to D_3_-induced differentiation, while the more resistant cell line (R38-) is only partially responsive to D_3_. We conclude that the defect in RA response is not necessarily compensated for by D_3_ treatment to enable myeloid differentiation, and the RA defect is apparently early and late, possibly reflecting dysfunctions in proper prolonged signaling during early and late stages. The signaling dysfunction notably involves reduced Fgr, c-Raf, and Vav1 expression. Overall, the results are of potential significance to the use of differentiation-inducing agents for overcoming RA resistance.

## Results

We examined if 1,25-dihydroxyvitamin D_3_ (D_3_) is able to upregulate differentiation markers in either R38+ or R38- retinoic acid (RA)-resistant HL-60 cells, and by exploiting the biphasic characteristics of HL-60 differentiation, whether D_3_ could compensate for RA response dysfunction during the precommitment (first 24 h) or lineage-commitment stage. HL-60 cells were treated with one inducer (RA or D_3_) for 24 h, then washed and retreated with either the same, different, or no inducer. The expectation was that the cells receiving the same differentiation agent will behave as if in continuous exposure, while the cells whose differentiation agent switched will reveal dependence of various differentiation markers on the precommitment vs. lineage-commitment stages. Cells receiving no retreatment will reveal what aspects of the differentiation program are precommitment-dependent and will provide a control for the retreated cases. For each treatment regimen, 1 µM RA or 0.5 µM D_3_ was used as these doses were previously shown to yield comparable levels of differentiation in HL-60.

### R38- exhibit diminished CD38 expression in response to D_3_ compared to R38+ and WT HL-60

**Figure 1:**
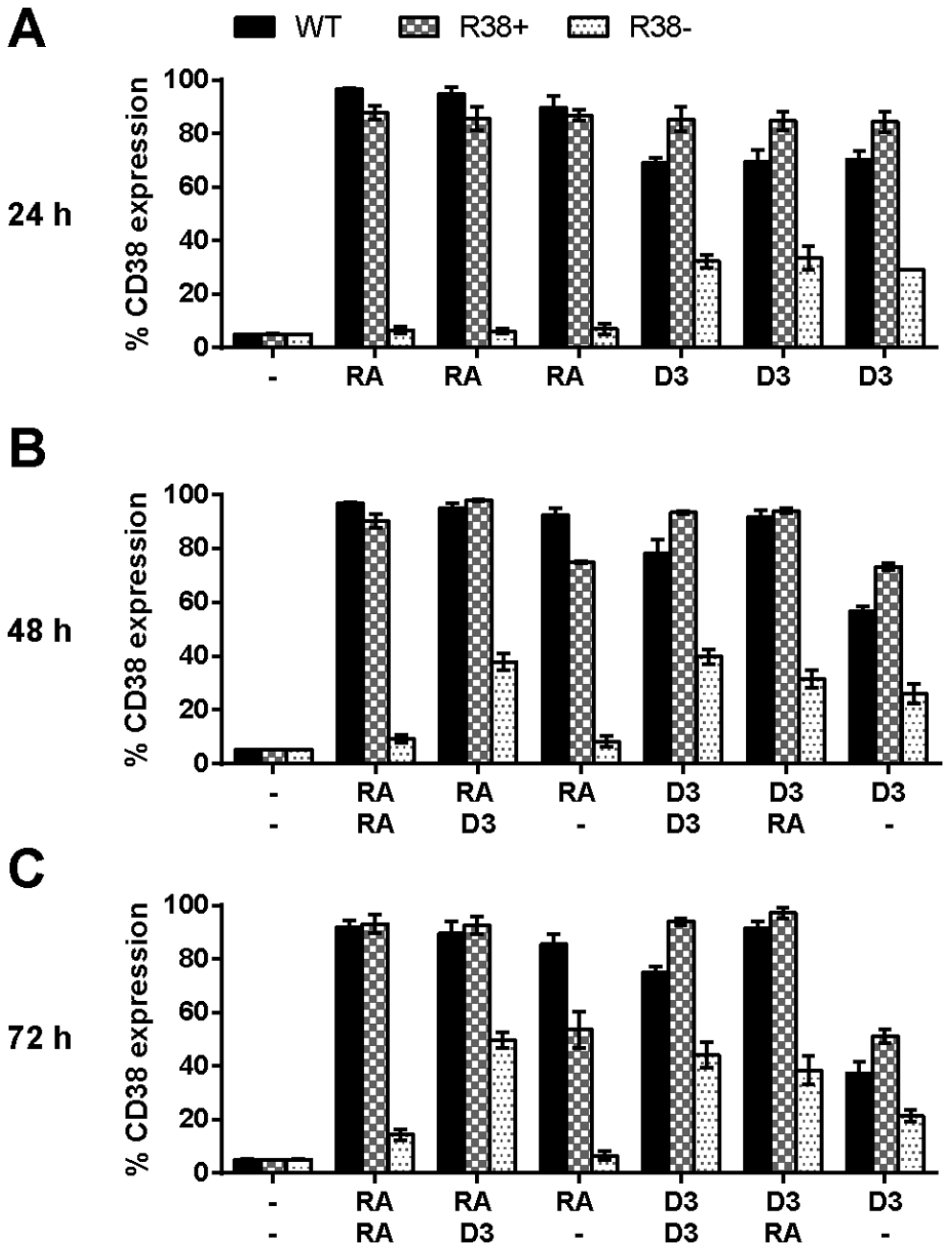
Percentage of cells expressing CD38 for WT HL-60 and R38+ and R38- RA-resistant HL-60 cells. D_3_ increases the early differentiation marker CD38 in RA-resistant HL-60 cell lines. (A) 24 h CD38 expression (after treatment with one inducing agent). (B) 48 h CD38 expression after sequential treatment with two inducing agents during the precommitment and lineage-commitment phases (RA/RA, RA/D_3_, RA/-, D_3_/D_3_, D_3_/RA, and D_3_/-). (C) 72 h CD38 expression (continuation of treatment with second inducing agent). CD38 expression was assessed by flow cytometry at 24, 48 and 72 h after first treatment initialization. Gates to determine percent increase of expression with treatment were set to exclude 95% of the control population. For clarity, p-values are not indicated above bars due to the existence of multivariate comparison between cell lines, treatments, and time. However, p-values of interest are mentioned specifically in the main text.

We assessed CD38 expression at 24 h to probe resistance in early or precommitment events prior to retreatment with a second inducer, and hence prior to the lineage-commitment phase. At 24 h, both RA and D_3_ induced CD38 expression in WT HL-60 (Fig. 1A). Three RA and D_3_ cases are shown since each served as the initial 24 h treatment for the subsequent timepoints. An average of 94% and 70% of the WT cells were CD38 positive for RA and D_3_ respectively. At 24 h, R38+ and R38- respond to RA-treatment as similarly reported for 48 h [19], with R38+ expressing, and R38- failing to express, CD38 after RA treatment. CD38 expression in RA-treated R38+ was similar to that of RA-treated WT HL-60, while the CD38 expression level in RA-treated R38- was similar to untreated WT HL-60, as expected. D_3_ induced CD38 expression in both resistant cell lines. In R38- the expression was about 50% of the expression in the WT cells, and in R38+ the expression was significantly (p<0.05) higher than in WT. CD38 expression in D_3_-treated R38+ cells is similar to CD38 expression in RA-treated WT HL-60, despite expression in RA-treated WT HL-60 significantly exceeding D_3_-treated WT HL-60 (p<0.0001). Thus R38- cells have precommitment resistance to D_3_ but R38+ cells do not, and R38+ actually have higher CD38 expression than D_3_-treated WT HL-60.

48 h and 72 h timepoints probed resistance in later events. 48 h post treatment (24 h in precommitment and 24 h in lineage-commitment stages), R38- cells treated with RA/D_3_ or D_3_/D_3_ have comparable CD38 expression levels (38% vs. 40%, Fig. 1B). R38- cells treated with D_3_/RA are 31% positive for CD38, indicating that in R38-, D_3_ presented early or late could elicit CD38 expression at comparable levels, although it was short of that of WT cells. By 72 h, CD38 expression induced by D_3_ in the resistant cells does not increase significantly (Fig. 1C). R38+ cells present two interesting behaviors. First, in the cells receiving RA in the precommitment phase and not receiving any differentiation agent in the lineage-commitment phase (RA/-), a decrease in CD38 expression is more abrupt in R38+ (75% at 48 h vs. 54% at 72 h) than in WT HL-60 (92% vs. 85%), showing an impaired ability of R38+ to maintain CD38 expression. Second, D_3_/D_3_ and D_3_/- treatments in R38+ have higher levels of CD38 than WT HL-60 cells (p <0.005 and p<0.05 respectively).

Overall, WT HL-60 cells behave as expected, with CD38 expression increasing over time during all treatment patterns, except for RA/- and D_3_/-, in which CD38 expression decreases as the cells revert to a proliferating state. R38+ have more CD38 expression than WT when treated with D_3_ first (but not with RA first), and show a more rapid decrease in CD38 expression than WT during RA/- and D_3_/-. R38- have half the induced CD38 expression compared to WT during RA/D_3_ and D_3_/D_3_, slightly less for D_3_/RA, no CD38 expression during RA/RA or RA/-, and decreasing CD38 expression during D_3_/-. Hence compared to R38+, R38- cells have a diminished response to D_3_ in terms of CD38 expression, and they have a more pronounced early defect not apparent in R38+.

### WT, R38+ and R38- HL-60 cells comparatively display decreasing D_3_-induced CD11b expression

**Figure 2:**
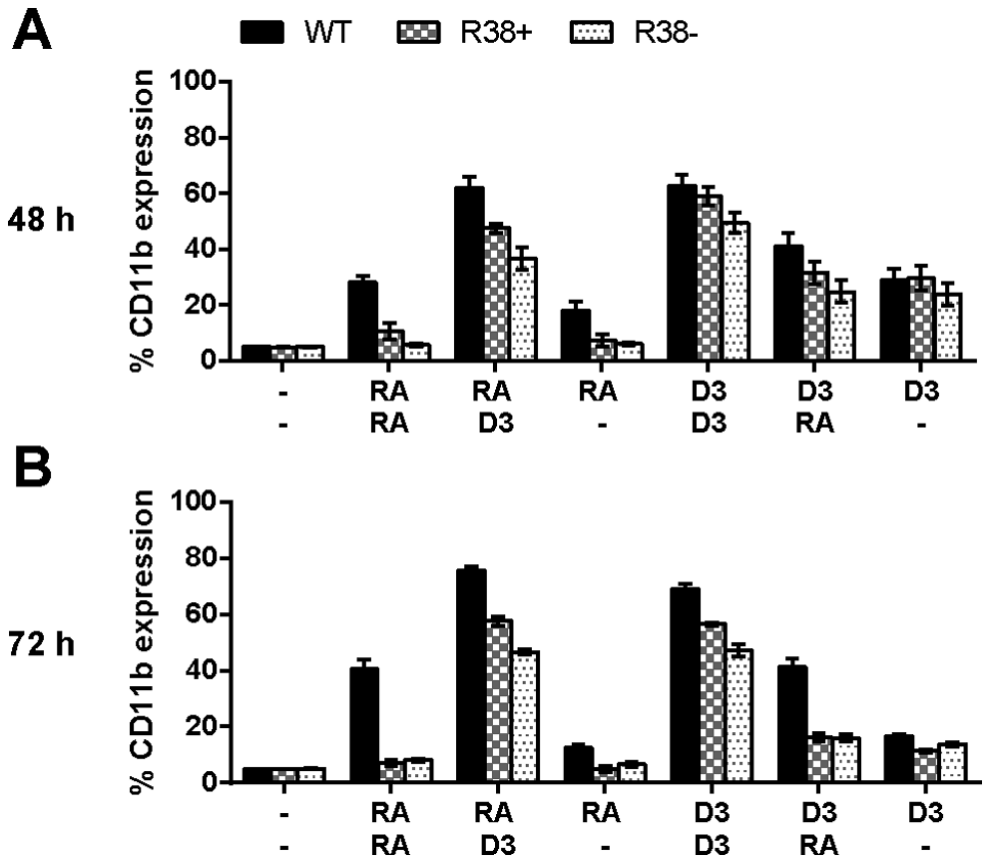
Percentage of cells expressing CD11b for WT HL-60 and R38+ and R38- RA-resistant HL-60 cells. D_3_ increases the differentiation marker CD11b in RA-resistant HL-60 cell lines. (A) 48 h CD11b expression after sequential treatment with two inducing agents during the precommitment and lineage-commitment phases (RA/RA, RA/D_3_, RA/-, D_3_/D_3_, D_3_/RA, and D_3_/-). (B) 72 h CD11b expression (continuation of treatment with second inducing agent). CD11b expression was assessed by flow cytometry (with APC-conjugated antibody) at 48 and 72 h after first treatment initialization. Gates to determine percent increase of expression with treatment were set to exclude 95% of the control population. For clarity, p-values are not indicated above bars due to the existence of multivariate comparison between cell lines, treatments, and time. However, p-values of interest are mentioned specifically in the main text.

CD38 is the only marker significantly expressed during the precommitment stage (first 24 h). We next focused on 48 h and 72 h to probe resistance in late events. CD11b is an integrin component expressed in granulocytes and monocytes [20]. RA does not induce CD11b expression in either RA-resistant HL-60 cell line [19]. D_3_ rescues CD11b expression in both R38+ and R38- cells when administered early or late, with R38+ being slightly more responsive (Fig. 2). The effect of D_3_ on CD11b expression appeared to be more potent if administrated during the lineage-commitment stage. Comparing RA/D_3_ and D_3_/RA at 48 h, p<0.004 for WT HL-60, p = 0.03 for R38+ and p = 0.01 for R38- (Fig. 2A). By 72 h, the D_3_/RA-treated R38+ and R38- cells have similar levels of CD11b to cells treated with D_3_/-, indicating that retreatment with RA was comparable to no retreatment for both the resistant cell lines (Fig. 2B). Overall, WT HL-60 behaved as expected. WT HL-60 exhibited increasing (over time) CD11b expression for all treatment regimens save for RA/- and D_3_/-, in which CD11b expression levels dropped by 72 h. Since D_3_/RA treatment, compared to D_3_/D_3_, did not fully restore a WT-like response in the RA-resistant cells, the data suggest a late RA defect(s), which is putatively more pronounced in R38- than R38+.

### D_3_-induced CD14 expression occurs in WT, R38+ and R38- HL-60 cells if administered during the lineage-commitment phase

**Figure 3:**
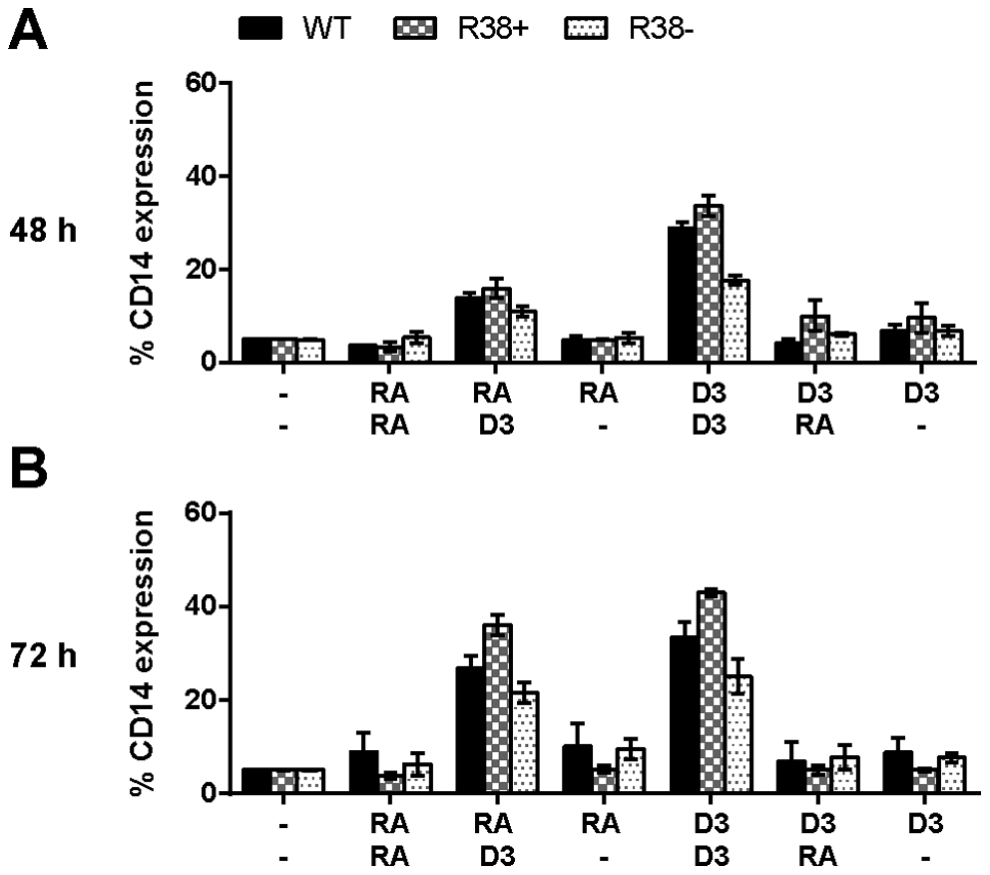
Percentage of cells expressing CD14 for WT HL-60 and R38+ and R38− RA-resistant HL-60 cells. D_3_ induced monocytic differentiation marker CD14 expression in wild-type (WT) and both RA-resistant cell lines. (A) 48 h CD14 expression after sequential treatment with two inducing agents during the precommitment and lineage-commitment phases (RA/RA, RA/D_3_, RA/-, D_3_/D_3_, D_3_/RA, and D_3_/-). (B) 72 h CD14 expression (continuation of treatment with second inducing agent). CD14 expression was assessed by flow cytometry (with PE-conjugated antibody) at 48 and 72 h after first treatment initialization. Gates to determine percent increase of expression with treatment were set to exclude 95% of the control population. For clarity, p-values are not indicated above bars due to the existence of multivariate comparison between cell lines, treatments, and time. However, p-values of interest are mentioned specifically in the main text.

CD14 is a glycosylphosphatidylinositol-anchored membrane protein expressed by monocytes, but not by granulocytes [21]. CD14 is a monocytic-specific marker for detecting a differentiation response to D_3_ treatment, and here its expression reveals whether defects are lineage unrestricted or restricted (i.e. early or late). We expect WT HL-60 cells to exhibit CD14 expression only during D_3_/D_3_ and RA/D_3_ treatments. By 72 h, all three cell lines treated with D_3_/D_3_ expressed CD14 at significantly higher levels than all other treatments, with R38+ expressing slightly higher levels (43% positive cells) than the WT HL-60 cells (33% positive cells, Fig. 3B). At 72 h there is no significant difference between RA/D_3_ and D_3_/D_3_ treated cells for either R38+ or R38− individually, although R38+ cells had higher CD14 expression than R38− cells (Fig. 3B). This indicates that monocytic differentiation can potentially occur in the RA-resistant cells, and that monocytic differentiation can occur if the differentiation agent present during the lineage-commitment phase is D_3_. The R38− response was weaker than the R38+ response, consistent with progressive attenuation of D_3_ response as cells become more resistant.

### D_3_ cannot rescue respiratory burst activity in either R38+ or R38−

**Figure 4:**
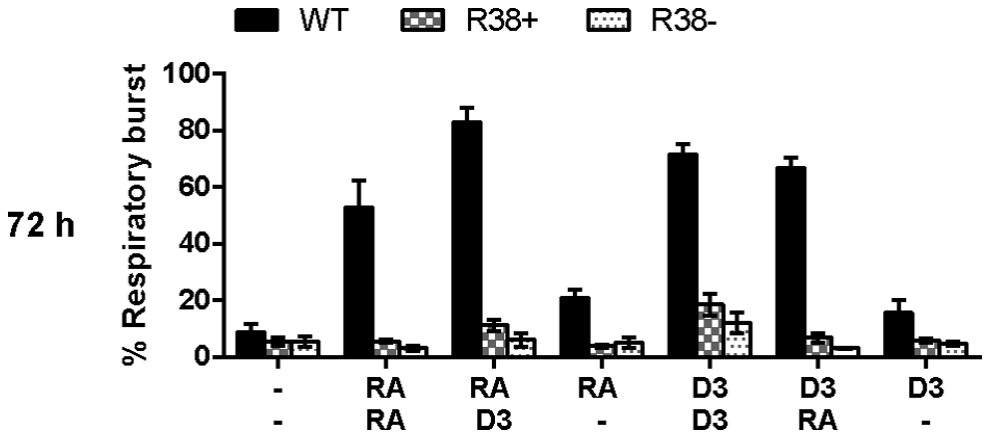
Percentage of cells exhibiting inducible respiratory burst for WT HL-60 and R38+ and R38- RA-resistant HL-60 cells. Oxidative metabolism (respiratory burst) at 72 h after sequential treatment with two inducing agents during the precommitment (24 h) and subsequent lineage-commitment phase (RA/RA, RA/D_3_, RA/-, D_3_/D_3_, D_3_/RA, and D_3_/-). Respiratory burst activity in RA-resistant HL-60 cells was only marginally rescued by D_3_ only when present both in precommitment and commitment stages of differentiation. WT HL-60 cells exhibit significant respiratory burst when RA or D_3_ is present during the lineage-commitment phase. Gates to determine percent increase of expression with treatment were set to exclude 95% of the DMSO control population.

Respiratory burst (oxidative metabolism) is the ability of mature neutrophils and macrophages to respond to bacterial infections, and is considered a final functional marker of maturity. None of the treatment regimens were able to significantly rescue this late differentiation marker in the RA-resistant HL-60 (Fig. 4, also see Discussion). Although D_3_/D_3_ treatment tended to increase the respiratory burst activity, this did not reach statistical significance. The WT HL-60 behaved as expected, exhibiting a strong respiratory burst in all treatment cases except for RA/- or D_3_/-.

### R38- cells comparatively display the lowest level of D_3_-induced G1/G0 cell cycle arrest

**Figure 5:**
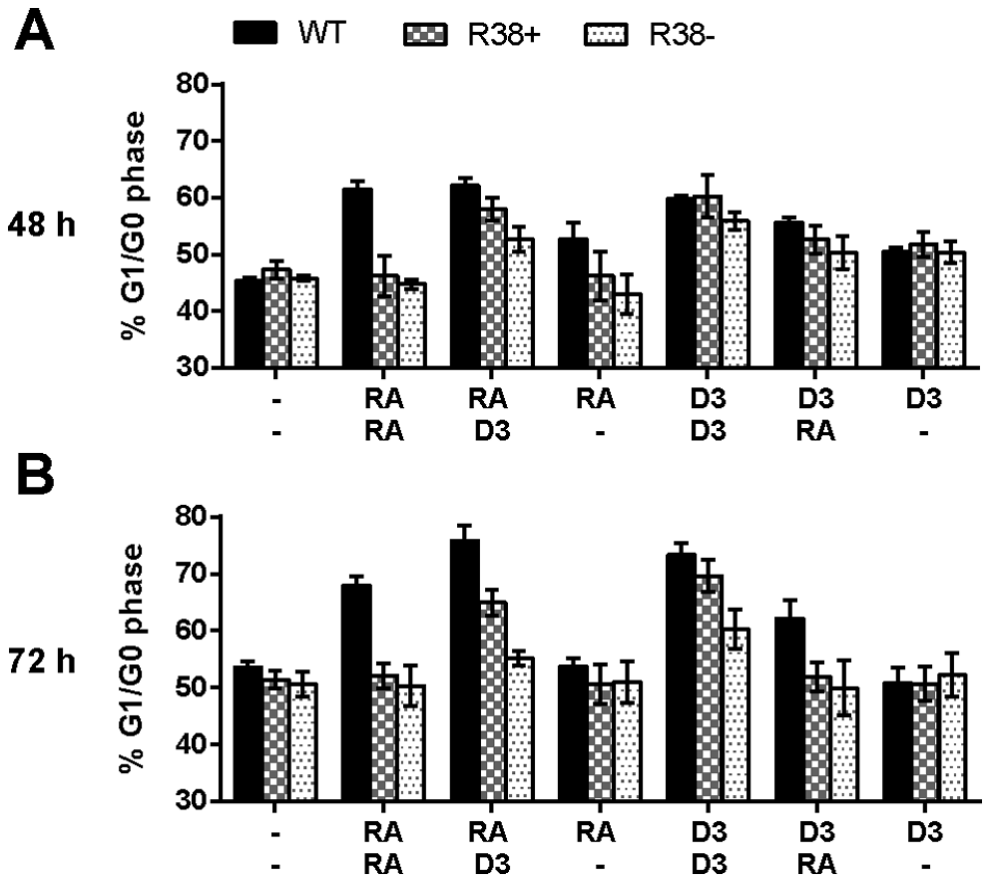
Percentage of cells in the G1/G0 phase for WT HL-60 and R38+ and R38- RA-resistant HL-60 cells. D_3_ rescued G1/G0 arrest in R38+, and to a lesser degree in R38-, when added in the lineage-commitment stage. (A) 48 h G1/G0 arrest after sequential treatment with two inducing agents during the precommitment and lineage-commitment phases (RA/RA, RA/D_3_, RA/-, D_3_/D_3_, D_3_/RA, and D_3_/-). (B) 72 h G1/G0 arrest (continuation of treatment with a second inducing agent). Untreated control gates were set to 45% G1/G0, 35% S and 20% G2/M. For clarity, p-values are not indicated above bars due to the existence of multivariate comparison between cell lines, treatments, and time. However, p-values of interest are mentioned specifically in the main text.

We examined G1/G0 cell cycle arrest, which is also a relatively late attribute of induced differentiation. In WT HL-60, all treatments except RA/- and D_3_/- induced a significant (p<0.005) G1/G0 enrichment at 48 h (Fig. 5A). RA-treated RA-resistant cells do not exhibit G1/G0 arrest. For both R38+ and R38-, the only treatments that significantly (p<0.05) increased the proportion of cells in G1/G0 were RA/D_3_ and D_3_/D_3._ Differences between the individual responses of R38+ and R38- were not yet significant at 48 h. By 72 h, the WT cells treated with RA/RA, RA/D_3_, D_3_/D_3_ or D_3_/RA were significantly (p<0.005) arrested in G1/G0 (Fig. 5B)_._ Also at 72 h, R38+ cells were arrested by RA/D_3_ and D_3_/D_3_ treatments, while the R38- resistant cells were arrested just by D_3_/D_3_ treatment (p = 0.03). Thus for the R38+ cells, D_3_ had to be administrated at least in the lineage-commitment stage, whereas for the more severely resistant R38- cells, D_3_ had to be administrated in both precommitment and lineage-commitment stages to obtain significant growth arrest by 72 h. This is consistent with a late differentiation dysfunction for R38+ and early and late dysfunction for R38-.

### D_3_ rescues early (24 h) expression of Fgr, Vav1, and p47^phox^ in RA-resistant cells

Significant signaling components that are upregulated during the first 48 h of RA treatment in HL-60 cells have been identified [8], [10], [22]–[29]. Knowing the behavior of this ensemble during RA-induced differentiation, we sought deviations from this in the resistant cells to gain insight into the potential molecular basis of the cellular phenotypic behavior above. We examined the CD38-associated proteins c-Cbl, Vav1, and Slp76; the Src-family kinases (SFKs) Lyn and Fgr, and the Y416 SFK phosphorylation site; c-Raf and its RA-induced phosphorylation sites S259, S621 and S289/296/301; p47^phox^, one of many proteins related to oxidative metabolism; and aryl hydrocarbon receptor (AhR) which we reported drives differentiation.

**Figure 6:**
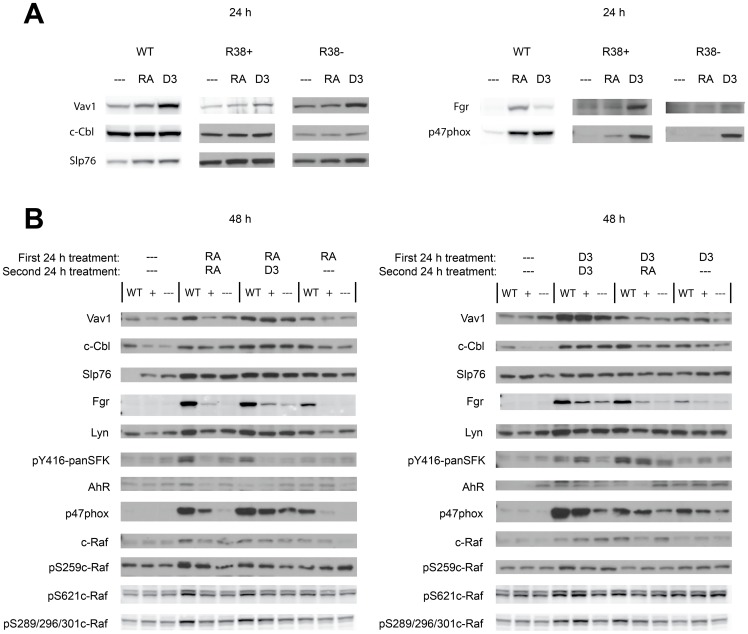
Signaling protein expression for WT HL-60 and R38+ and R38− RA-resistant HL-60 cells. Individual Western blots of whole cell lysates are representative of at least three repeats. GAPDH loading controls were also performed on each individual blot to ensure even loading (not shown). WT HL-60 samples are indicated by WT, R38+ samples are indicated by + and R38− samples are indicated by ---. (A) 24 h protein expression after treatment with a single inducer (RA or D_3_) during the precommitment phase. (B) 48 h protein expression after sequential treatment with two inducing agents during the precommitment and lineage-commitment phases (RA/RA, RA/D_3_, RA/-, D_3_/D_3_, D_3_/RA, and D_3_/-). Quantified blot data are presented in Figure 7.

**Figure 7:**
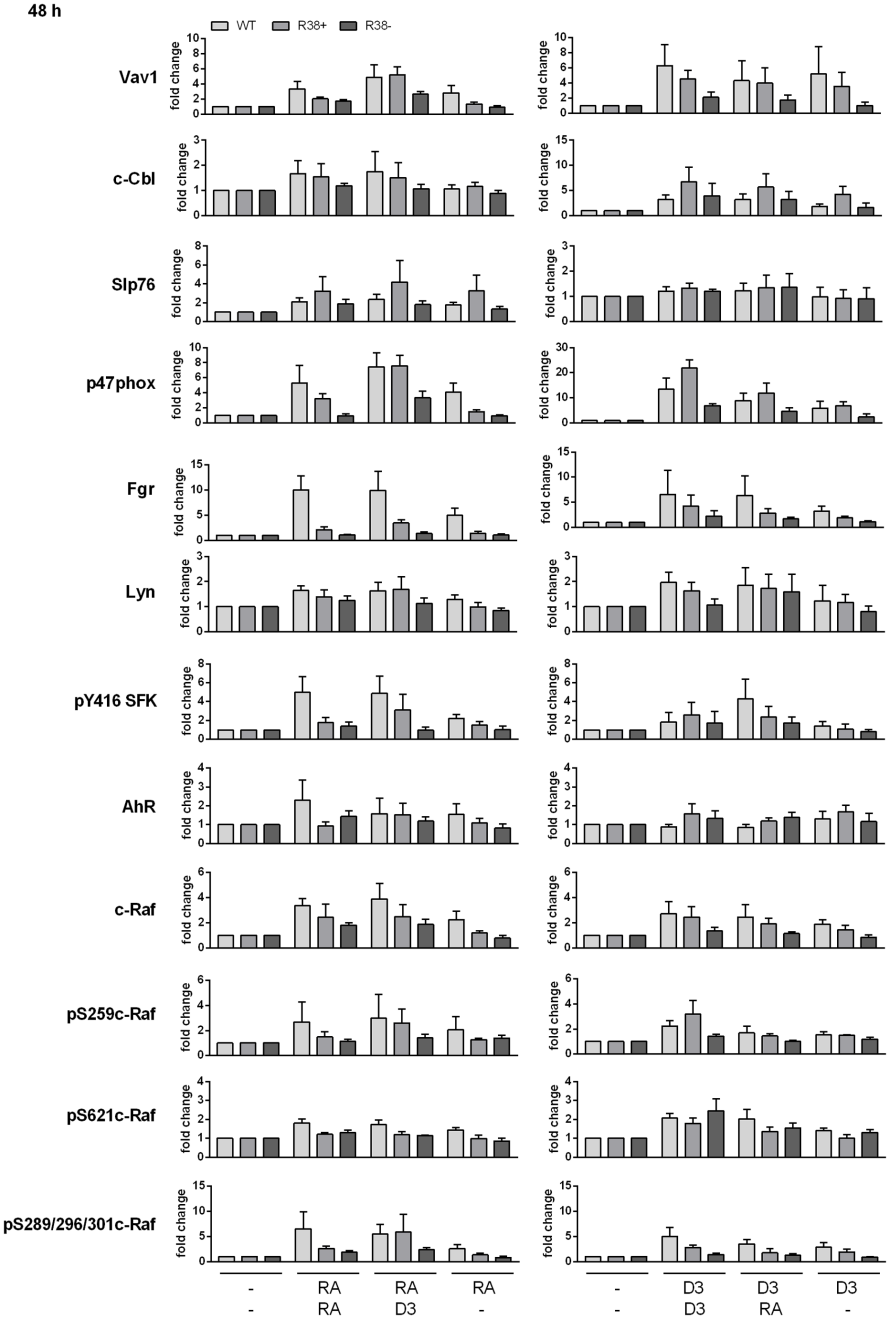
Quantified 48-60 and R38+ and R38− RA-resistant HL-60 cells. Repeat 48Figure 6 were quantified using ImageJ and average fold change from control was graphed in GraphPad. Error bars represent standard error. Note that the fold change axis scale may differ for each bar graph.

We first investigated proteins known to exhibit increased expression in WT HL-60 by 24 h. Fgr is upregulated by RA after 24 h in WT HL-60, but not in R38+ or R38- resistant cells. Fgr could still be upregulated by D_3_ in R38+ cells at 24 h.(Fig 6A). But R38− cells were slower and required 48 h (Fig 6B, Fig 7) before Fgr upregulation was discernible. This correlates with the putative more profound resistance of R38− cells. Vav1 is upregulated by RA and D_3_ in WT HL-60, and in contrast to Fgr, D_3_ results in higher Vav1 expression during the first 24 h compared to RA treatment. But in resistant cells, RA did not cause any appreciable Vav1 upregulation, whereas D_3_ is able to increase Vav1 expression in both RA-resistant cell lines. p47^phox^ is comparably induced by RA and D_3_ in WT HL-60 during the first 24 h. But in R38+ cells RA induces only a small increase in p47^phox^ and none detectable in R38−. In contrast D_3_ treatment prominently increases expression of p47^phox^ in both RA-resistant lines. Slp76 is upregulated by both RA and D_3_ in WT HL-60, but expression in resistant cells hardly changed with either RA or D_3_ treatment at 24 h. Meanwhile c-Cbl does not exhibit much change by the end of the early 24 h timepoint. There are thus early defects in RA-induced upregulation of select signaling molecules such as Fgr, Vav1, and p47^phox^, whose expression in the resistant cells is, to some degree, rescued by D_3_.

### WT, R38+ and R38− HL-60 cells comparatively display decreasing D_3_-induced expression and phosphorylation of differentiation-associated signaling factors at 48 h

We examined signaling factor expression at 48 h to probe for resistance-related aberrations during the late, lineage-commitment phase. Representative blots for 48 h signaling data are shown in Fig. 6B. To address slight variations across repeats, all repeats where quantified and average fold change ± S.E.M. is presented in Fig. 7. Also, Figure S1 regraphs the same data separated by cell line to further clarify the expression differences. At 48 h, WT HL-60 treated with RA/RA behaved as expected, with RA exposure resulting in increased Fgr, Lyn, Vav1, c-Cbl, Slp76, c-Raf, AhR and p47^phox^ expression (Fig. 6B, Fig. 7) compared to untreated control. However, RA-resistant cells (R38+ and R38−) treated with RA/RA displayed little to no upregulation of these signaling proteins (excluding Slp76, as found previously [19]) compared to WT HL-60. For the RA/D_3_ treatment case, WT HL-60 again show upregulated expression of these proteins. Interestingly, R38+ and R38− treated with RA/D_3_ exhibited increased Vav1 and p47^phox^ expression compared to the RA/RA case, although expression in R38− was diminished relative to R38+. R38+ cells also exhibited c-Cbl expression with RA/D_3_ treatment, but R38− tended not to. Lyn and c-Raf were minimally increased in the RA-resistant cells during RA/D_3_ treatment. In the resistant cells, Fgr expression is only slightly increased during the RA/D_3_ case, with the R38+ line exhibiting less Fgr expression compared to WT HL-60, and R38− even less compared to R38+. Overall this indicates that D_3_ treatment, despite the previous RA treatment, was able to predominantly rescue expression of Vav1 and p47^phox^ signaling proteins in R38+ and less so in R38−, consistent with the more apparent resistance to D_3_ of R38− compared to R38+. As most evidenced by Fgr, WT cells treated with RA/- (i.e. RA followed by no retreatment) displayed similar but diminished expression of signaling proteins compared to the RA/RA case, consistent with a need for continuous early and late exposure to drive expression.

For D_3_/D_3_ treated cells, WT and R38+ lines displayed upregulated Fgr, Lyn, Vav1, c-Cbl, c-Raf and p47^phox^ expression compared to untreated controls, while R38− had notably diminished expression of these proteins, again consistent with greater D_3_ response dysfunction in R38− compared to R38+ (Fig 6B, Fig 7). For the D_3_/RA treatment pattern, WT HL-60 still show upregulation of these proteins. But for the RA-resistant R38+ and R38− cells, D_3_ followed by RA treatment resulted in less Fgr, Vav1, c-Cbl, c-Raf and p47^phox^ expression compared to the D_3_/D_3_ case, consistent with a putative late defect in both resistant cell lines.

During RA/RA treatment in WT HL-60, phosphorylation at c-Raf sites S259, S621, and S289/296/301 is increased (Fig. 6B, Fig. 7). For the resistant cells, this response was largely lost. For RA/D_3_ treatment, the pS259c-Raf and pS289/296/301c-Raf responses were recovered in R38+ cells, but not in R38− cells, consistent with their putative greater resistance. In both resistant cells, the pS621c-Raf response was lost in RA/D_3_ treatments, consistent with the importance of this phosphorylation event in driving differentiation as suggested earlier [30]. D_3_/D_3_ treatment induced phosphorylation at all c-Raf sites checked in WT and R38+ cells. Both R38+ and R38− retained S621c-Raf during D_3_/D_3_ treatment, but unlike R38+, phosphorylation at S259 and S289/296/301 sites on c-Raf was lost in R38− cells. Increased loss of induced c-Raf phosphorylation thus correlated with increased resistance. c-Raf phosphorylation was generally reduced in D_3_/RA-treated RA-resistant cells compared to the D_3_/D_3_ case. Overall these c-Raf phosphorylation changes did not necessarily correlate with the changes in c-Raf expression level. In the different treatment regimens, RA and D_3_, administered singly or in sequential combination, caused increased c-Raf expression in WT HL-60. The response in each instance was diminished in R38+ and even more diminished in R38−. The Y416 SFK (Src-family kinase) site also showed increased phosphorylation in RA-treated WT HL-60 cells, and this response was largely abrogated in both resistant cells. D_3_ administered early or late tended to cause, albeit much smaller, an increase in Y416 SFK phosphorylation in the R38+ but not R38− cells.

**Figure 8:**
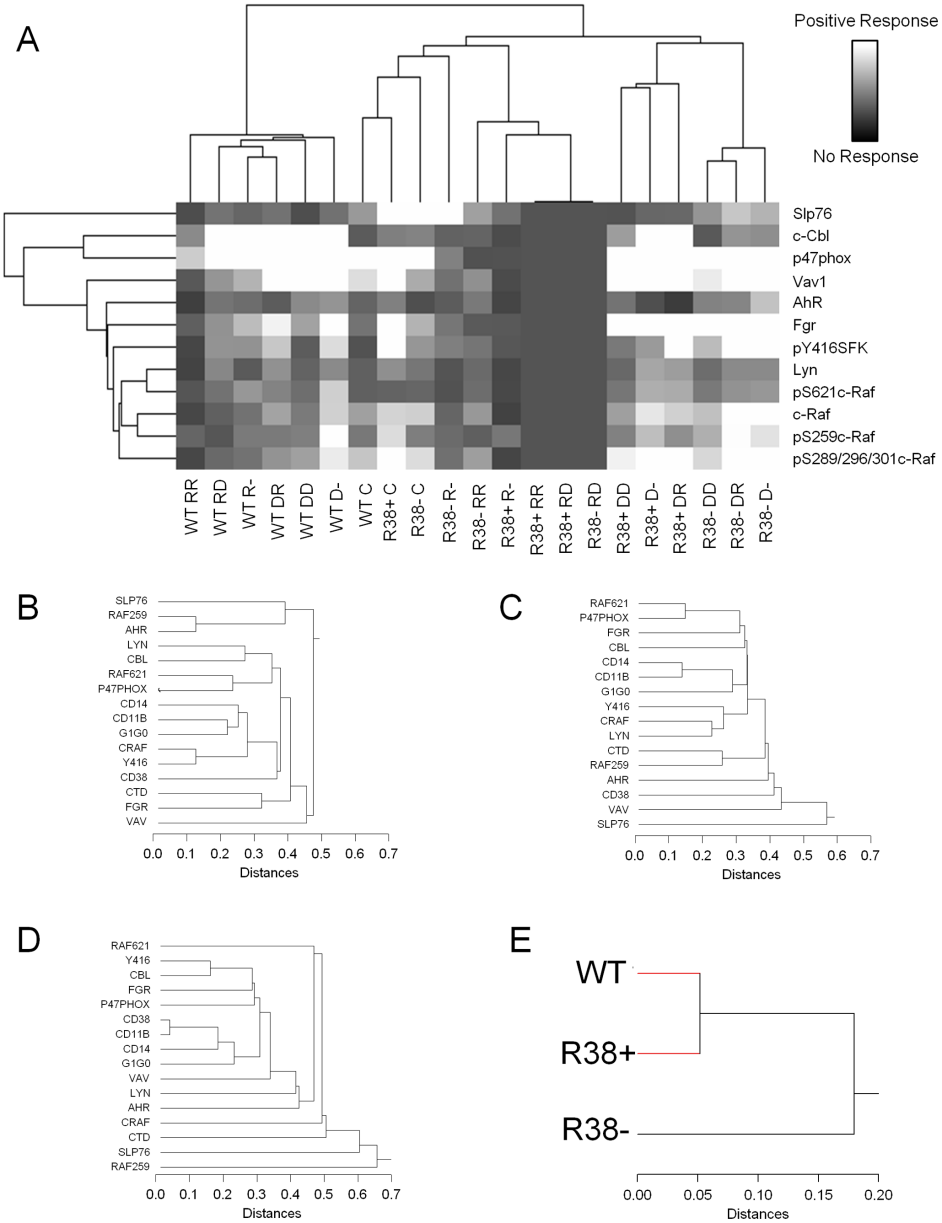
Clustering analysis. (A) Agglomerative hierarchical clustering analysis was performed on average quantified Western blot data (signaling protein data only) using Cluster 3.0 and visualized with TreeView. The distance metric was Pearson's correlation coefficient. In the diagram, RR  =  RA/RA, RD  =  RA/D_3_, R-  =  RA/-, DD  =  D_3_/D_3_, DR  =  D_3_/RA, and D-  =  D_3_/-. (B-E). Clustering analysis across all treatment cases and all results (cytometry phenotyping data and Western blot signaling protein data) was performed using SYSTAT 8.0. In B-E, CTD refers to pS289/296/301c-Raf. (B) Clustering for WT HL-60. (C) Clustering for R38+. (D) Clustering for R38−. (E) WT and R38+ cluster more closely to each other than to R38− HL-60 cells.

Taken together the above data motivate the notion that this ensemble of signaling molecules and events support differentiation and that progressive resistance is concomitant with their decreased expression and phosphorylation. Cluster analysis (see Discussion) reveals that in WT HL-60 cells there is a tight coupling between the responses of the ensemble of signaling molecules for different treatment regimens, but the coupling is degraded as the cells become progressively more resistant (Figs. 8B-D).

## Discussion

### Phenotypic Differences between WT, R38+ and R38− HL-60 cells

To our knowledge, this is the first study that analyzes how RA resistance depends on early vs. late lineage-commitment events in lineage-bipotent myeloid cells and relates to lineage cross-resistance. Taking the cellular response data together, the responses of R38+ and R38− RA-resistant HL-60 cells to the combinatorial sequences of RA and D_3_ treatment distills to two basic results. First, in both R38+ and R38− resistant cells, D_3_/RA treatment does not restore RA response, while RA/D_3_ could not fully restore D_3_ response. Thus D_3_ cannot necessarily abrogate the temporally segregated early or late RA defect(s). Second, as the resistance to RA became more pronounced with progression from R38+ to R38−, there was progressive emergence of worse D_3_ response. That is, the response to D_3_ administered early or late in combination with RA, or administered both early and late, was less effective in R38− than R38+ cells. Although both R38+ and R38− cells equally failed to develop a significant oxidative metabolism, D_3_ treatment can nevertheless rescue expression of the respiratory burst-associated protein p47^phox^ in the resistant cells, with the greatest expression occurring during D_3_/D_3_ treatment and slightly lower during RA/D_3_ treatment, and greater expression always occurring in R38+ compared to R38−. When treated with D_3_ during the lineage-commitment phase, R38+ cells always exhibited higher CD14 expression than R38− cells. R38− cells consistently displayed lower CD38 and CD11b expression, lower differentiation-associated signaling factor expression and phosphorylation, and notably had lower G1/G0 cell cycle arrest compared to both WT and R38+ HL-60. Therefore we found that RA differentiation therapy resistance can develop in stages, with initial partial RA resistance and moderate D_3_ responsiveness (unilineage maturation block), followed by subsequent pronounced RA resistance and partial D_3_ resistance (bilineage maturation block).

RA can inhibit monocyte/macrophage activity [31], and other differentiation programs can also be suppressed by a RAR-dependent process [32]. In the case of WT HL-60 cells, although the precommitment stage can be induced by RA or D_3_, the later stages of monopoiesis are inhibited by RA [15], [16]. If enhancing the differentiation process toward one lineage may inhibit another, then it may be plausible that cells resistant to one induced lineage can respond more strongly to another induced lineage (i.e., the “repressive” pathway is removed). This could be one explanation for why early D_3_ treatment induced a slightly stronger response in the R38+ RA-resistant cells than the WT cells in terms of CD38 and CD14 expression.

We performed hierarchical clustering analysis between the cell lines across all treatments and results, and interestingly found that WT and R38+ clustered more closely than R38− (Fig. 8E). Agglomerative hierarchical clustering analysis across all cell lines and treatments vs. signaling components is diagramed in Fig. 8A. The treatment cluster family for WT HL-60 separates into two clusters: those treated with RA first and those treated with D_3_ first. The untreated control samples exist in a cluster with R38− RA/RA and R38− RA/-. This is consistent with the notion that R38− is the most resistant cell line and consequentially the least dissimilar from untreated WT cells. Allowing that R38− RA/- represents the least responsive case, then the cluster analysis reveals a progression of cases that become more distal to and deviate from the most unresponsive case, namely R38− RA/−, R38− RA/RA, R38+ RA/−, R38+ RA/RA, and finally the RA/D_3_ cases for both resistant lines. This clustering conforms to the anticipation that R38− are less responsive than R38+, and that RA is generally less effective than D_3_ in eliciting response in the resistant cells. The cases for early D_3_-treated resistant cells group together further away in the clustering analysis, consistent with weaker resistance to D_3_ compared to RA posited earlier.

When comparing both the signaling results and the cellular phenotypic results, hierarchical clustering across all treatments for WT (Fig. 8B), R38+ (Fig. 8C), and R38− (Fig. 8D) reveals the increasing distances (lower correlations) as cells become more resistant compared to the WT HL-60 cells. A progressive uncoupling of the signaling molecules thus occurs as WT HL-60 change to R38+ and then to R38−. Thus the repertoire of signaling proteins surveyed may have a seminal role in effecting differentiation. Progressive degradation of the clustering of an ensemble of putative signalosome molecules as resistance increases supports the importance of an intimate co-regulated clustering of those molecules to drive differentiation. We investigated RARα and VDR protein levels at 24 and 48 h (Figure S2) and were unable to attribute decreasing resistance to loss of either receptor.

### Vav1, Fgr and c-Raf emerge as prominent differentiation-associated factors

A potential suite of molecular dysfunctions is seminal to the progression of observed resistance phenotypes. Vav1 is required for RA-induced granulocytic differentiation [33] as well as TPA-induced monocytic differentiation of HL-60 [34]. Vav1, along with c-Cbl and Slp76, exhibit increased expression and exist in a CD38-associated complex during RA-induced differentiation of WT HL-60 [9]. These signaling factors are also upregulated along with CD38 during D_3_ treatment in WT HL-60, as well as in RA-resistant HL-60. A cohort of molecules known to interact with CD38 is evidently expressed along with CD38 during either monocytic or granulocytic differentiation.

D_3_ induced Vav1 expression in R38+ and R38− during the first 24 h. If the two RA-resistant lines were retreated with D_3_, then Vav1 expression persisted. However if the second treatment was RA, Vav1 expression tended to diminish by 48 h. A similar outcome occurs for c-Cbl, and p47^phox^. Thus, although ectopic overexpression of Vav1 or c-Cbl can enhance RA-induced differentiation in WT HL-60 [9], [33], early-induced expression of these signaling factors in resistant cells is not enough to propel RA-induced differentiation during the lineage-commitment stage, which may reflect the co-existence of other potential defects. The data suggest that a late Vav1-dependent function may be disrupted in resistance, and lesser Vav1 expression in R38− compared to R38+ cells may contribute to the increased D_3_ resistance in R38− cells.

We have previously reported that the Src-family kinases (SFKs) Lyn and Fgr are upregulated with RA treatment in WT HL-60 cells [10]. The D_3_-induced upregulation of Lyn and Fgr has been noted by us and others [35]. Lyn and Fgr are the predominant SFKs expressed in myeloid cells [35], [36]. However of the two, only Lyn appears to be the predominantly active (phosphorylated) kinase in RA-induced HL-60 [10], [37], as well as in RA-treated NB4 cells [38]. Lyn and Fgr have been found to exert their functional roles in distinct subcellular compartments [38]. When the aryl hydrocarbon receptor (AhR) ligand 6-formylindolo(3,2-b)carbazole (FICZ) enhances Lyn and Fgr expression, as well as Vav1, c-Cbl, and p47^phox^ expression, it also enhances RA-induced differentiation in WT HL-60 cells [39].

In R38+ and R38− RA-resistant HL-60 cells, Fgr expression was not induced by RA at 24 h, as expected, yet was only minimally rescued either early or late by D_3_ compared to WT HL-60 cells. Thus Fgr may be a signaling component important to the non-resistant phenotype, and dysfunctional early RA regulation of Fgr emerges as a prominent feature of resistance that correlates with loss of cellular phenotypic response. Phosphorylation at the Y416 SFK site seems to be primarily an RA-driven event in the WT HL-60 cells, as the highest pY416 SFK phosphorylation occurs during RA/RA, RA/D_3_ and D_3_/RA treatments. In contrast, results for Lyn and AhR were not as striking. Overall, there appeared to be higher Lyn expression in WT HL-60 cells across all treatment patterns. But upregulation by RA/RA, RA/D_3_, D_3_/D_3_ or D_3_/RA for all cells lines remained similar; hence it is not apparent whether Lyn expression is specific to any inducing agent or phase of differentiation. Similar results were obtained for AhR, with the exception of RA/RA treated WT HL-60, which had the highest AhR expression among all treatment cases and cell lines.

c-Raf phosphorylation appears disrupted in resistance. Phosphorylation at the putative inhibitory site S259, the stability site S621, and the functionally ambiguous S289/296/301 site has been found to be induced by RA [10], [19], [30]. Here we show that S259 c-Raf phosphorylation may be an early (but not late) RA driven event. Enhanced pS259c-Raf is observed in WT cells during RA/RA, RA/D_3_, and RA/− treatment (Fig. 7A), but not during D_3_/RA treatment (despite higher phosphorylation for the D_3_/D_3_ case). Also p259c-Raf is increased in the R38+ HL-60 during RA/D_3_ and D_3_/D_3_, but not D_3_/RA, treatment. Thus for both the WT and R38+ cells, pS259 was higher during RA/D_3_ than D_3_/RA treatment, consistent with being an early RA-driven event. Interestingly, the phosphorylation site S289/296/301 was significantly increased (as high as in WT cells) in R38+ during RA/D_3_ treatment, but when D_3_ was used first, R38+ had less pS289/296/301c-Raf than WT (Fig. 7A and 7B).

Overall for c-Raf expression and phosphorylation, the R38− cells tended not to show as great a response to D_3_ compared to WT or R38+, whether D_3_ was treated first or last (Fig. 7C). This is again indicative of the greater degree of disrupted c-Raf-dependent signaling in these cells. Consistent with this, c-Raf expression was similarly progressively less during all treatments across WT, R38+ and R38− HL-60 cells. Like Vav1 and Fgr, c-Raf emerges as putatively a key component of the non-resistant phenotype. p47^phox^ and c-Cbl expression may be correlated with CD38 and/or CD14, since these two signaling factors were also more highly expressed in early D_3_-treated R38+ cells compared to WT HL-60. But Fgr, Vav1 and c-Raf showed decreasing (across WT, R38+, R38−) induced expression for all treatments, similar to CD11b expression and G1/G0 arrest, notably implicating their dysfunction in progressive resistance.

### D_3_ treatments and RA resistance in other studies

Retinoic acid (RA) and the active form of vitamin D_3_, 1,25-dihydroxyvitamin D_3_ (D_3_), are dietary factors that demonstrate chemotherapeutic efficacy in inducing maturation in leukemia cells. RA is the current treatment for acute promyelocytic leukemia (APL) [40], and retinoids serve preventative and therapeutic roles in other cancers and diseases [3], [41], [42]. D_3_ is able to exert anti-proliferative effects in other myeloid cells [43] and other cancer cell types [44]. It has been shown that analogs of D_3_ can induce differentiation of myeloid cells with minimal calcium toxicity [45]–[47]. Like D_3_, D_3_ analogs have shown efficacy in inducing differentiation not only in myeloid lines, but in prostate and breast cancer cells [48], [49]. Co-administration of RA with D_3_ or analogs thereof is a potential therapeutic strategy to mitigate the side effects of each individual inducer (RA syndrome, hypercalcemia, RA or D_3_ resistance).

One group found that RA and analogs of D_3_ can act synergistically in WT HL-60 to promote differentiation and inhibit cell growth [50], and that a RA-resistant HL-60 cell line is more sensitive to D_3_ treatment than the parental WT cells [51]. We previously developed an HL-60 cell line resistant to sodium butyrate (a monocytic inducer) that was also cross-resistant to RA; however, this line remained responsive to monocytic differentiation by D_3_ [52]. Interestingly, D_3_ was shown to induce granulocytic (not monocytic) differentiation in a RA-resistant APL cell line [53]. In another case, D_3_ treatment did not induce differentiation in RA-resistant HL-60 [6]. RA resistance in HL-60 has been historically attributed to mutation of RARα [54], [55]. However, in some RA-resistant myeloid lines where a mutation in RARα was found, expression of wild-type RARα did not fully restore RA responsiveness [6], [7]. It is clear that other defects arise, which most likely vary across resistant sublines developed in different laboratories. This may account for the varying reports seen in the literature regarding a response (or lack of response) of RA-resistant cells to D_3_ treatment. In fact, in a single study one group developed two RA-resistant HL-60 cell lines, one of which was D_3_-responsive and harbored a RARα mutation, while the other was D_3_-resistant and had intact RARα [56].

## Conclusions

The present study shows that induced signaling and phenotypic conversion progressively degrade in discernible stages of resistance. We showed that D_3_ cannot necessarily abrogate temporally segregated early or late RA-resistance defect(s). Nonetheless, D_3_ can induce extensive, but not complete, functional monocytic differentiation in the RA-resistant cells compared to WT HL-60. Therefore although the segregation of unilineage vs. bilineage resistance is not exact, we show that an RA-resistant cell line that retains partial RA-responsiveness (R38+) is more amenable to D_3_-induced differentiation, while a sequentially emergent cell line more resistant to RA (R38−) is less responsive to D_3_. We found that the emergent R38− RA-resistant HL-60 cell line was more dissimilar from WT and R38+ than WT and R38+ where from each other. An ensemble of signaling molecules that are co-regulated in WT HL-60 become progressively more uncoupled as resistance becomes more pronounced, a trend involving increasing loss of response to RA and then D_3_. There was a putative early Fgr expression dysfunction and a late Vav1-dependent dysfunction correlated with progressive resistance, as well as dysfunctional c-Raf expression. HL-60 are negative for the t(15;17) mutation, making RA-induced mechanisms in these cells potentially applicable to other cancers. Overall RA resistance may thus result from dysfunction of multiple pathways, rather than single genetic defects.

## Materials and Methods

### Cell culture and treatments

HL-60 human myeloblastic leukemia cells, derived from the original patient isolates, were a generous gift of Dr. Robert Gallagher, and were maintained in this laboratory and published previously ([8]–[17] and others). HL-60 wild-type (WT), and the two RA-resistant HL-60 (R38+ and R38−) cells subsequently isolated in our laboratory [19] were grown in RPMI 1640 supplemented with 5% fetal bovine serum (both: Invitrogen, Carlsbad, CA) and 1x antibiotic/antimycotic (Sigma, St. Louis, MO) in a 5% CO_2_ humidified atmosphere at 37°C. Cells were cultured in constant exponential growth as previously described [57]. Viability was monitored by 0.2% trypan blue (Invitrogen, Calsbad, CA) exclusion and routinely exceeded 95%. Experimental cultures were initiated at a density of 0.2×10^6^ cells/ml.

There were seven treatment regimens studied: (1) untreated, (2) RA/RA, (3) RA/D_3_, (4) RA/−, (5) D_3_/D_3_, (6) D_3_/RA, and (7) D_3_,/−. The first agent, RA or D_3_, was added for the first 24 h (precommitment phase) followed by wash and retreatment with either the same, different, or no inducing agent (−) for the second 24 h (lineage-commitment phase) and beyond, for a total of 48 and 72 h. After 24 h of initial treatment, cultures underwent two washes of 10 min each in 15 ml of RPMI 1640 supplemented with 5% fetal bovine serum and 1× antibiotic/antimycotic before resuspension in fresh complete media and retreatment. The reported results indicate the total timepoint, encompassing both prewash and postwash treatments. All-*trans* retinoic acid (RA) (Sigma, St. Louis, MO) was added from a 5 mM stock solution in 100% ethanol to a final concentration of 1 µM in culture. 1,25-dihydroxyvitamin D_3_ (D_3_) (Cayman, Ann Arbor MI) was added from a 1 mM stock solution in 100% ethanol to a final concentration of 0.5 µM in culture. All other reagents were purchased from Sigma (St Louis, MO) unless otherwise indicated.

### CD38, CD11b and CD14 quantification

1×10^6^ cells were collected from cultures and centrifuged at 700 rpm for 5 min. Cell pellets were resuspended in 200 µl 37°C PBS containing 2.5 µl of either APC-conjugated CD11b antibody, PE-conjugated CD38 antibody, or PE-conjugated CD14 antibody (all from BD Biosciences, San Jose, CA). Following 1 h incubation at 37°C, cell surface expression levels were analyzed with a BD LSRII flow cytometer (BD Biosciences, San Jose, CA). APC fluorescence (excitation at 633 nm) was collected with a 660/20 band pass filter and PE fluorescence (excitation at 488 nm) was collected with a 576/26 band pass filter. Undifferentiated control cells were used to determine the fluorescence intensity of cells negative for the respective surface antigen. The gate to determine percent increase of expression was set to exclude 95% of the control population.

### Respiratory burst quantification

1×10^6^ cells were collected and centrifuged at 700 rpm for 5 min. Cell pellets were resuspended in 500 µl 37°C PBS containing 5 µM 5-(and-6)-chloromethyl-2′,7′-dichlorodihydro–fluorescein diacetate acetyl ester (H_2_-DCF, Molecular Probes, Eugene, OR) and 0.2 µg/ml 12-o-tetradecanoylphorbol-13-acetate (TPA, Sigma, St. Louis, MO). Both, H_2_-DCF and TPA stock solutions were made in DMSO at concentrations of 0.2 mg/ml and 5 mM, respectively. A control group incubated in H_2_-DCF and DMSO without TPA was included. Cells were incubated for 20 min at 37°C prior to analysis by flow cytometry. Oxidized DCF was excited by a 488 nm laser and emission collected with a 530/30 nm band pass filter. The shift in fluorescence intensity in response to TPA was used to determine the percent cells with the capability to generate inducible oxidative metabolites [58]. Gates to determine percent positive cells were set to exclude 95% of control cells not stimulated with TPA.

### Cell cycle quantification

1×10^6^ cells were collected by centrifugation at 700 rpm for 5 min and resuspended in 200 µl of cold propidium iodide (PI) hypotonic staining solution containing 50 µg/ml propidium iodine, 1 µl/ml Triton X-100, and 1 mg/ml sodium citrate. Cells were incubated at room temperature for 1 h and analyzed by flow cytometry using 488-nm excitation and collected with a 575/26 band-pass filter. Doublets were identified by a PI signal width versus area plot and excluded from the analysis [58], [59].

### Protein detection by Western Blotting

2×10^7^ cells were lysed using 350–400 µL lysis buffer (Pierce, Rockford, IL) supplemented with protease and phosphatase inhibitors (Sigma, St. Louis, MO), and lysates were cleared by centrifugation at 13,000 rpm for 30 min at 4°C. Equal amounts of total protein lysates (15 µg) were resolved by SDS-PAGE, transferred onto PVDF membranes and probed with antibodies. c-Cbl (C-15) antibody was from Santa Cruz Biotechnology (Santa Cruz, CA). pS621c-Raf antibody was from Pierce Thermo Scientific (Lafayette, CO). Lyn, Fgr, pY416-SFK, AhR, Vav1, Slp76, p47^phox^, c-Raf, pS259c-Raf, pS289/296/301c-Raf, VDR, RARα, GAPDH, horseradish peroxidase anti-mouse and horseradish peroxidase anti-rabbit were from Cell Signaling (Danvers, MA, USA). Enhanced chemiluminescence ECL reagent (GE Healthcare, Pittsburg, PA) was used for detection.

### Statistical analysis

Treatment group means were compared using the Paired-Samples T-Test. The data represent the means of three repeats ± S.E.M. A p-value of <0.05 was considered significant (using GraphPad software and Excel). For agglomerative hierarchical clustering of signaling data, average quantified Western blot data was clustered using Cluster 3.0 and visualized with TreeView. To assess the correlation of the expression patterns of both the phenotypic markers and signaling molecules, a hierarchical cluster analysis [60] was conducted by single linkage method (nearest neighbor) and marker similarity metrics based on the Pearson correlation using SYSTAT 8.0 software.

## Supporting Information

Figure S1 **Quantified 48 h protein expression for WT HL-60 and R38+ and R38- RA-resistant HL-60 cells graphed separately.** Repeat 48 h Western blot data were quantified using ImageJ and average fold change from control was graphed in MATLAB. Each cell line is graphed separately for comparison. Error bars represent standard error. (A) WT HL-60 cells, all treatments and signaling proteins. (B) R38+ HL-60 cells, all treatments and signaling proteins. (C) R38- HL-60 cells, all treatments and signaling proteins. The fold change axis scale is maintained for each graph.
(TIF)

Figure S2 **Quantified 24 h and 48 h RARα and VDR expression for WT HL-60 and R38+ and R38- RA-resistant HL-60 cells.** Three repeats of Western blot data (using whole cell lysates) were quantified using ImageJ and average fold change from control was graphed in GraphPad. Error bars represent standard error. GAPDH loading controls were also performed on each individual blot to ensure even loading (not shown). Note that the fold change axis scale may differ for each bar graph. (A) At 24 h there is minimal to no change in the expression of RARα or VDR with RA or D_3_ treatment in all three cell lines. (B) Interestingly RA tended to increase VDR expression in WT HL-60 cells at 48 h. R38+ cells tended to have slightly higher receptor expression when treated with D_3_ first. However, overall we found that increasing resistance of any cell line could not be attributable to significant loss of either receptor.
(TIF)
