# Supplementary figures and images for: Retinoic Acid Therapy Resistance Progresses from Unilineage to Bilineage in HL-60 Leukemic Blasts
Source: PLoS One. 2014 Jun 12;9(6):e98929. doi: 10.1371/journal.pone.0098929 (PMC4055670; doi:10.1371/journal.pone.0098929)

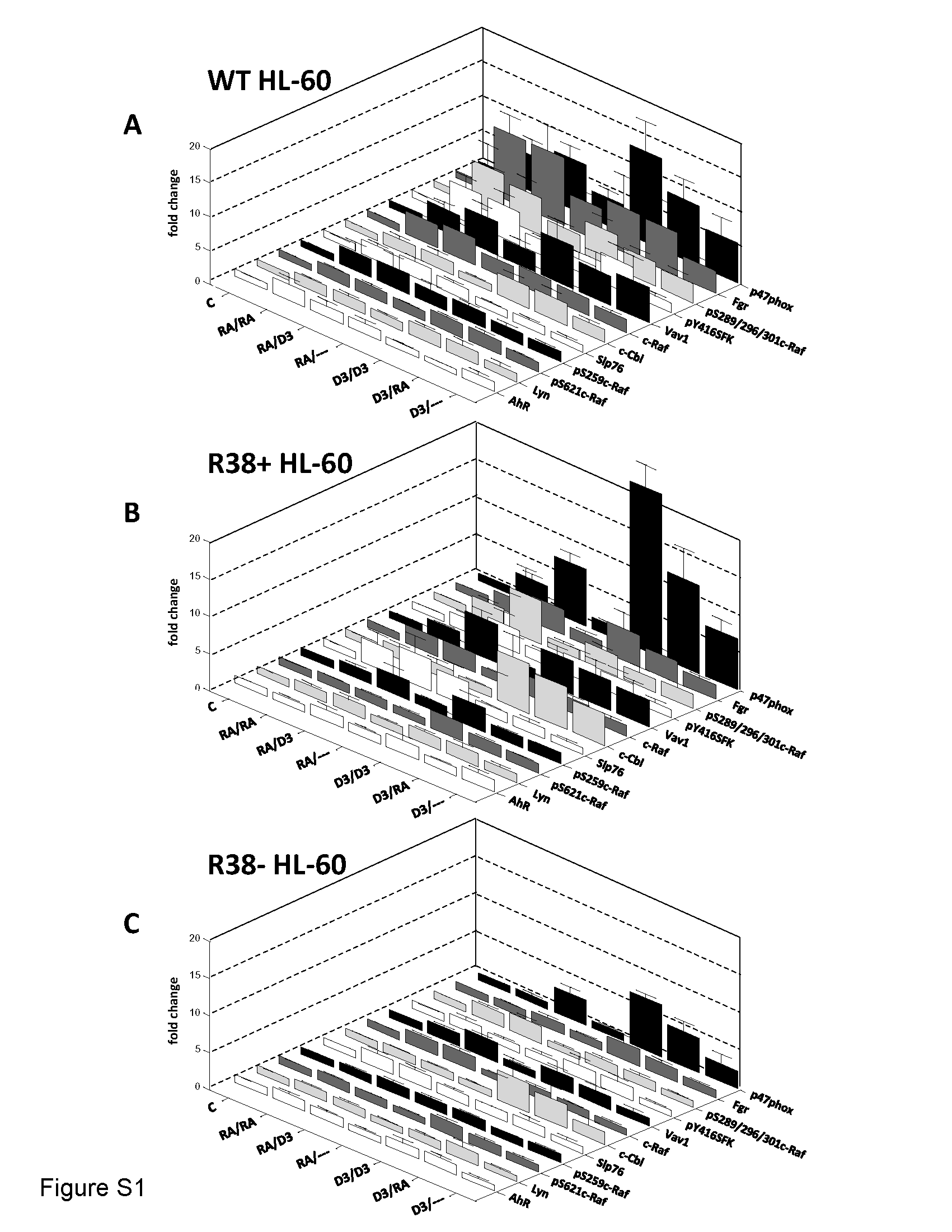

Supplement: Figure S1 — Quantified 48 h protein expression for WT HL-60 and R38+ and R38- RA-resistant HL-60 cells graphed separately. Repeat 48 h Western blot data were quantified using ImageJ and average fold change from control was graphed in MATLAB. Each cell line is graphed separately for comparison. Error bars represent standard error. (A) WT HL-60 cells, all treatments and signaling proteins. (B) R38+ HL-60 cells, all treatments and signaling proteins. (C) R38- HL-60 cells, all treatments and signaling proteins. The fold change axis scale is maintained for each graph. (TIF) [file pone.0098929.s001.tif]
